# Recent advances of pathomics in colorectal cancer diagnosis and prognosis

**DOI:** 10.3389/fonc.2023.1094869

**Published:** 2023-07-19

**Authors:** Yihan Wu, Yi Li, Xiaomin Xiong, Xiaohua Liu, Bo Lin, Bo Xu

**Affiliations:** ^1^ School of Medicine, Chongqing University, Chongqing, China; ^2^ Chongqing Key Laboratory of Intelligent Oncology for Breast Cancer, Chongqing University Cancer Hospital, Chongqing, China; ^3^ Bioengineering College, Chongqing University, Chongqing, China

**Keywords:** artificial intelligence, deep learning, machine learning, colorectal cancer, pathomics

## Abstract

Colorectal cancer (CRC) is one of the most common malignancies, with the third highest incidence and the second highest mortality in the world. To improve the therapeutic outcome, the risk stratification and prognosis predictions would help guide clinical treatment decisions. Achieving these goals have been facilitated by the fast development of artificial intelligence (AI) -based algorithms using radiological and pathological data, in combination with genomic information. Among them, features extracted from pathological images, termed pathomics, are able to reflect sub-visual characteristics linking to better stratification and prediction of therapeutic responses. In this paper, we review recent advances in pathological image-based algorithms in CRC, focusing on diagnosis of benign and malignant lesions, micro-satellite instability, as well as prediction of neoadjuvant chemoradiotherapy and the prognosis of CRC patients.

## 1 Introduction

Colorectal cancer (CRC) is the third most commonly diagnosed cancer and the second-leading cause of cancer-related deaths globally, according to the Global Cancer Statistics 2020 (1). The 5-year survival rate for CRC varies from 14% for distant-stage patients to 90% for localized-stage patients (2). As such, accurate diagnosis and prognosis prediction are crucial for improving the survival rate of patients (3–6). Despite recent advances of our understanding on the mechanisms driving CRC tumorigenesis, using multi-omics data for accurately predicting the CRC prognosis with high accuracy are still far reaching.

After years of rapid development, Artificial intelligence (AI) based algorithms have evolved from traditional machine learning (7, 8) to complex deep learning (9–11), with the latter being especially adept at identifying complex features in medical images, including radiology images (such as those from CT and MRI scans) and pathology images (10). Thanks to whole slide image (WSI) scanners, digital pathology is now possible, allowing traditional pathological slides to be converted into digital images for permanent storage. WSIs contain complex information – large sizes (10,000 x 10,000 pixels), color information (H&E and immunohistochemistry), and multiple magnifications (10X, 20X, 40X) (12). The digitalization of pathological images has facilitated the transmission of image-rich pathological data between distant locations (13) and has been widely used in digital diagnosis, remote consultation, education, and research (14). The performance of computer-based algorithms for digital WSIs diagnoses of cancer has almost reached that of experienced pathologists (15, 16). Furthermore, some algorithms can predict the status of molecular markers (17, 18), identify genetic mutations responsible for cancer (19, 20), determine treatment responses (21, 22), and predict survivals (23, 24). These researches highlight the potential of AI to extract comprehensive and sub-visual information from routine pathological images. On the basis of these studies, the concept of pathomics has emerged (25), which converts pathological images into mineable datasets based on AI algorithms and links these extracted and quantified pathological features to clinically related indicators. Researchers have explored applications of AI-based pathological image analyses and achieved satisfactory results in many cancers, especially in CRC.

In this review, we will discuss the workflow of pathomics and their advances in CRC.

## 2 Pathomics workflow

**Figure 1:**
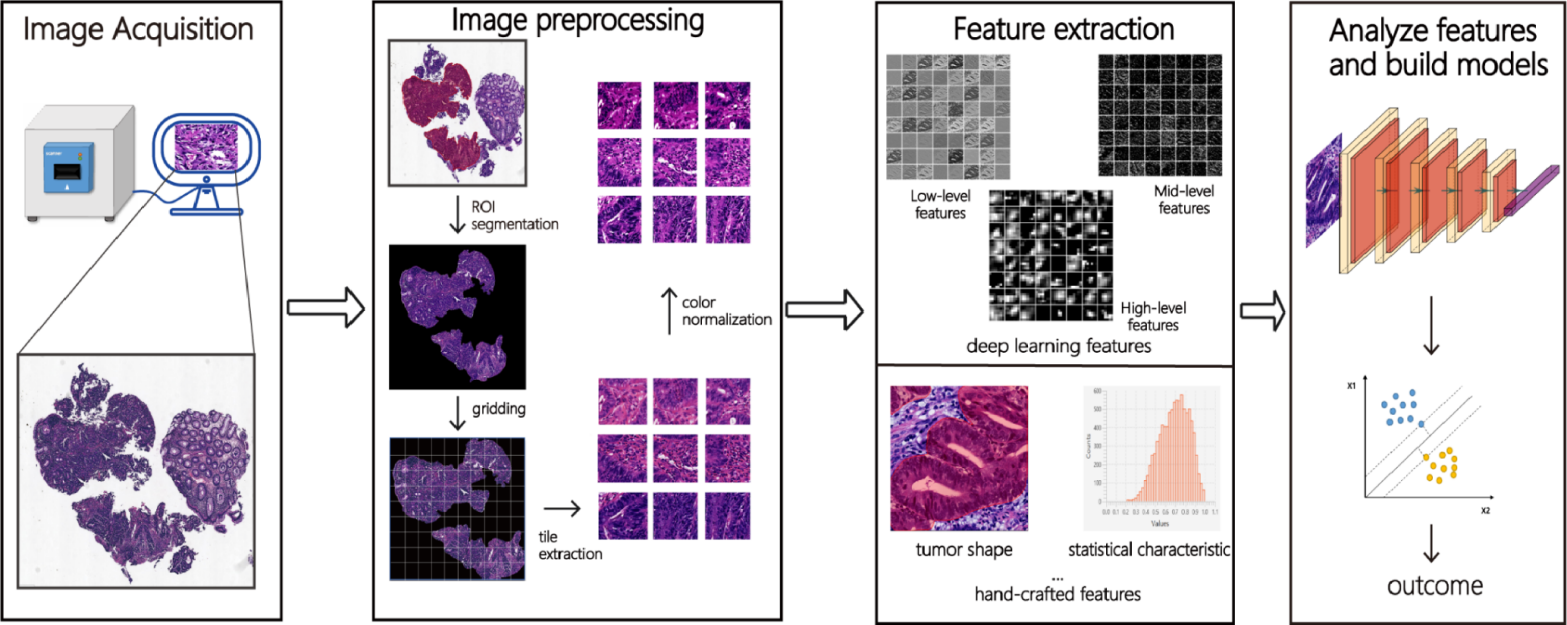
The pathomics workflow. Firstly, after collecting and scanning pathological images, the ROI (region of interest) is manually or automatically labeled. Secondly, deep learning features (low-level, mid-level, and high-level features) and hand-crafted features (morphology, texture, statistics, and other features) are extracted from these images through a series of images pre-processing such as ROI segmentation, gridding, tile extraction, and color normalization. Finally, meaningful features are analyzed by machine learning or deep learning algorithms and classified or predicted according to different tasks.

The pathomics analysis workflow consists of three main steps: the selection of regions of interest (ROIs), color normalization, and the extraction and analysis of pathomics features. **Figure 1** illustrates a typical pathomics workflow.

### 2.1 Selection of ROIs

The initial step in pathomics analysis involves outlining regions of interest (ROIs) on a whole slide image (WSI) to identify areas that require processing or analysis, such as tumor and interstitial regions. Processing the whole WSI is computationally intensive, time-consuming, and may incorporate irrelevant or confusing information. Only defining the ROI enables narrowing down the image analysis to the most pertinent parts, which reduces computational costs and enhances the quality of analysis. Furthermore, defining the ROI allows the extraction of representative and distinctive features, which assists with identifying, classifying, or predicting disease states. Defining the ROI allows for the extraction of representative and distinctive features, leading to improved model performance. Thus, effective ROI outlining and appropriate tile extraction are significant factors to be considered in the analysis of pathological images.

ROI outlining methods include manual or automatic delineation. Professional pathologists generally use dedicated software such as Qupath (26) and ASAP (27) for manual delineation, which is accurate and flexible but time-consuming, labor-intensive, subjective, and not repeatable. As such, auxiliary tools have been developed to enhance the efficiency and accuracy of manual methods. Automatic methods involve using algorithms to achieve automatic or semi-automatic ROI drawing. This method involves pre-processing the image, identifying and locating ROIs using specific algorithms. Automation can save human resources, enhance consistency and repeatability, and adapt to large-scale data processing. However, automatic methods may not effectively handle image quality differences, complex backgrounds, and varied target morphology. To improve the performance and robustness of automatic methods, tissue classifiers (28–30) have been proposed for automatic classification, which have shown reasonable overall performance. Public databases such as NCT-CRC-HE-100K (31) (100,000 image tiles) and CRC-VAL-HE-7K(7180 image tiles) (31) are available for training CRC classification models. There are various ROI outlining schemes that have their own advantages and disadvantages. Selecting appropriate methods based on various scenarios and needs, combining artificial intelligence and professional knowledge, is crucial to achieve efficient and accurate ROI delineation.

### 2.2 Color normalization

During the preparation of colorectal tissue sections, there are inevitable color variations in WSIs, even with the same staining protocol, among different laboratories, which limits the generalization power of an algorithm. Factors causing color variations include the difference in dyeing time, concentration and pH of staining solutions, staining platforms, and scanner models (32). Several CRC-related studies (28, 33) evaluated the impact of color variations on model efficiency and found that models built with color normalization achieved higher efficacy than those without normalization. Therefore, researchers have proposed various normalization techniques to reduce the impact of image color variations on the training models. Currently, there are two main categories of color normalization methods: statistics-based and physical model-based. Statistics-based methods aim to match the color space of images to the statistical features of a target image or standard image. For example, Reinhard et al. (34) put forward a linear normalization method in lαβ color space by balancing the mean and standard deviation of each dimension. However, this method ignores the color difference in different areas of the image (such as the background and different dyes). To solve this problem, Khan et al. (35) proposed an automatic segmentation and Gaussian mixture model method for normalizing the color of each region. Physical model-based methods establish mathematical models of the color formation process in pathological images and use inversion or optimization techniques to calculate dye concentration or absorption coefficients. For example, Ruifrok and Johnston (36) proposed a method based on Lambert-Beer law and matrix decomposition, which transformed RGB images into dye concentration space, and normalized or de-stained them. This method can better retain information on tissue structure, but it is necessary to know or estimate the absorption spectrum of dyes in advance. Recently, some researchers have explored the use of neural networks, such as Cycle-GAN (37) for normalizing the color of pathological images, which adapts automatically to different types and sources of images and generates realistic and diverse results.

### 2.3 Extraction and analysis of pathomics features

The objective of pathomics feature extraction is to transform complex, high-dimensional, and diverse image data into simplified, low-dimensional feature vectors. There are traditional and deep learning methods for feature extraction. Traditional methods require expert knowledge to design and select suitable feature descriptors, including first-order features (such as shape, size, texture, and color distribution) and second-order features (features obtained by calculating intermediate matrices and defining a series of statistics, such as the color histogram and the gray co-occurrence matrix).

These hand-crafted features are utilized in machine learning models, such as Support Vector Machine (SVM) and random forests, for tumor classification and prognosis analysis (38–40). However, these techniques are dependent upon pre-existing knowledge and expertise, and may not be able to capture high-level and abstract information. In recent years, deep learning methods have gained popularity due to their ability to automatically learn feature representation based on neural network models, such as convolutional neural networks (CNN). These methods adaptively extract abstract and high-level features from a large number of pathological images and optimize features and classifiers simultaneously. The deep learning method has been shown to outperform traditional methods in pathological image analysis (41, 42), discovering features that have not been recognized by humans. However, as network layers become deeper, extracted features are more heavily abstracted and frequently lack explanations surrounding individual dimensions (13). Some studies (43–45) have presented a method that combines traditional and deep learning features. The combination of these features has been shown to produce improved detection accuracies than traditional or deep learning features utilized separately.

Over-fitting may occur due to the high dimensionality and potential redundancy of features extracted from pathological images. Therefore, feature selection and dimension reduction techniques can be leveraged to identify the most representative and predictive features. Standard dimension reduction techniques include Principal Component Analysis (PCA) and Linear Discriminant Analysis (LDA). PCA is an unsupervised learning method used to project high-dimensional data into a lower-dimensional space while preserving the variance of the original data. Conversely, LDA is a supervised learning method that maps samples to a low-dimensional space to maximize the differences between categories. Once feature selection and dimension reduction are complete, machine learning algorithms such as logistic regression, decision trees, support vector machines, and deep learning algorithms can be implemented to model pathological images and predict disease risk or diagnosis. The choice of algorithm is dependent on the data’s nature and the target task requirements, with decision trees being suitable for models that need to be explained and deep learning algorithms for high-precision models. Apart from predicting disease risk and diagnosis, analyzing the relationship between selected features and diseases can also shed light on the pathogenesis and treatment methods of diseases. Correlation analysis, cluster analysis, factor analysis, and machine learning algorithms are commonly used analytical methods for this purpose. By identifying relevant characteristics and biomarkers, we can better understand the disease’s pathogenesis and develop effective treatment plans.

## 3 Recent advances of pathomics in CRC diagnosis

### 3.1 Identification of CRC cells

The early detection and accurate diagnosis of CRC are crucial for reducing mortality rates. Numerous studies have demonstrated the diagnostic potential of pathomics in detecting CRC, and the summary of these studies is presented in **Table 1**. The data used in the current research are from The Cancer Genome Atlas (TCGA) Program’s public database and private datasets of hospitals. The Area Under the ROC Curve (AUC) and Accuracy (ACC) were the primary parameters used to evaluate the model’s performance.

**Table 1:** Literature overview of AI-based algorithms for CRC identification using histopathological images.

Reference	Number of cases	Number of classes	Ground truth	ROIs annotated method	Color Normalization	Modeling method	Result
Wang et al. (46)	N=14,234 (Patient n=6,876)	2	Cancer vs. non-cancer	manually	The color of each pixel was centered by the mean of each image and its range was converted/normalized from [0, 255] to [− 1, 1].	Inception V3	patch-level: ACC: 0.948-0.961 AUC: 0.983-0.985 patient-level: ACC: 0.934-0.990AUC: 0.911-0.992
Noorbakhs et al. (47)	N=27,815 (including 23 cancer types)	2	Cancer vs. normal tissue	manually	NA	Inception V3	ACC: 0.910 AUC:0.60-0.98
Feng et al. (48)	N=1,000 (patient n=600)	2	Benign vs. malignant	manually	NA	U-Net, VGG	Online test dataset: DSC:79.45% AUC: 1
Menon et al. (49)	N=9,297 (including 11 cancer types)	2	Cancer vs. normal tissue	NA	The mean and standard deviation of all RGB channels on the training set were calculated for normalization	ResNet-18	Self-organ: ACC: 0.920-0.99 Cross-organ: ACC: 0.530-0.980 AUC:0.525-0.998
Togacar et al. (50)	N=25,000 (including lung and colon cancers)	2	Adenocarcinoma vs. benign tissue	manually	NA	DarkNet-19	ACC: 0.997
Yu et al. (51)	N=13,111 (patient n=8,803)	2	Cancer vs. non-cancer	manually	The grayscale of each pixel was normalized to [-1,1]	Inception V3	Patch level: AUC:0.980 Patient level: AUC:0.974

For example, Wang et al. (46) developed an AI approach using transfer learning and the Inception-V3 CNN architecture to classify normal and cancerous tiles. The group collected 14,234 CRC WSIs from 6,876 patients in multiple institutions across China, the USA, and Germany, dividing them into four datasets for training and evaluation. This model achieved an AUC of 0.998 and an ACC of 0.981 at the tile-level prediction, reaching the highest ACC of 0.990 and AUC of 0.991 at the patient-level prediction. Meanwhile, the performance of the AI approach is comparable to professional pathologists with an AUC of 0.988 and 0.970, respectively. Based on the Inception V3 CNN architecture, Noorbakhsh et al. (47) trained a deep learning model for pan-cancer classification with an AUC of 0.995 and ACC of 0.910. 19 cancer subtypes can be classified, with AUCs ranging from 0.600 to 0.980. In addition to the model established based on the Inception architecture, some studies used VGG (48) and Res-Net (49) network to construct deep learning models for identifying benign and malignant lesions, with improved ACCs and AUCs. For example, the VGG-16 (48) model has achieved an AUC of 1 on the online test dataset of 250 HE-stained WSIs from 150 patients. In addition to the above-mentioned transfer learning, training an entire network from scratch can improve the performance of the model. For instance, Togacar et al. (50) used the DarkNet-19 model trained from scratch and the SVM method to detect the benign, malignant, and histological lung and colon cancer types, and utilized Equilibrium and Manta Ray Foraging optimization algorithms to choose efficient features. The ACC of the model after feature screening was higher than that of the model without feature screening.

For the small amount of labeled data, Yu et al. (51) proposed mixing training with a large amount of unlabeled data. Specifically, they used 13,111 WSIs collected from 8,803 CRC patients from 13 independent centers to develop a semi-supervised learning model (SSL, based on the mean teacher method, where the student and teacher models both used the Inception-V3 structure). They evaluated the SSL by comparing the performance of the SSL with SL (the supervised learning model, based on Inception-V3) and six professional pathologists. The performance levels of SSL and SL are similar at the tile level, with the AUCs of 0.980 and 0.987, respectively. The performance of SSL was comparable to that of the pathologists with the AUC of SSL, SL, and pathologists being 0.974, 0.980 and 0.969, respectively. In addition, the SSL was also confirmed in two other cancer types (lung cancer and lymphoma), indicating that the SSL can achieve similar performance as SL with massive annotations.

Su et al. (52) proposed a method to train the model for classification in overlapping pathological images using IHC as molecular markers of tumor regions on HE images. They developed an H&E molecular neural network (HEMnet) approach for automatically aligning HE images with corresponding IHC images. They used transfer learning to establish a VGG16-based CNN for classifying tiles as cancer or non-cancer. They selected TP53 as a biomarker from IHC, a critical tumor suppressor gene highly positive for staining in 74% of CRCs. TP53 positive regions in IHC images were labeled as tumor regions and registered with HEMnet at the same location in HE images, trained, and tested the model’s efficacy. Finally, the AUC for predicting p53 staining status was 0.730, and the AUC for predicting tumor regions annotated by pathologists was 0.840.

Convolutional neural networks are one of the commonly used deep learning algorithms among researchers diagnosing colorectal cancer using AI. AI has the potential to significantly enhance the accuracy of colorectal cancer diagnosis. This accuracy is consistent and unbiased and is not influenced by the experience of pathologists. In summary, AI-based colorectal cancer diagnosis holds great promise in the analysis of pathological images, with numerous avenues for exploration in the future, such as employing more advanced algorithms like deep reinforcement learning or performing comparisons and validation on multiple datasets.

### 3.2 Prediction of gene mutations

Mutation in several genes, including APC, TP53, RAS, BRAF, MLH1, MSH2, and MSH6, are associated with CRC (53). Among them, MLH1, MSH2, and MSH6 belong to the Mismatch Repair (MMR) system. When the MMR system is defective, the length of the microsatellite changes, resulting in microsatellite instability (MSI) (54). The MSI is a clinically important tumor marker and an essential molecular biomarker in almost all solid tumors (55), present in 10%-20% of CRC patients (56). The status of the MSI can provide information on prognosis and guide treatment. At present, most of the studies on pathological images are focused on the assessment of the microsatellite status, and some are involved in the prediction of other gene mutations.

**Table 2** summarizes the publications that report using different deep learning methods to predict microsatellite states. These models exhibit robust performance, with area under the curve (AUC) values ranging from 0.74 to 0.96 (28, 33, 57–62), and many outperform professional pathologists. For instance, Yamashita et al. (57) established MSINet, a model based on modified MobileNetV2, which produced stable performance. MSINet achieved an AUC of 0.865 on a TCGA dataset of 40 cases, compared to the average AUC of 0.605 of five pathologists. This indicates that the deep learning model is fully capable of reaching or even surpassing the human level.

**Table 2:** Literature overview of AI-based algorithms for gene mutations using histopathological images.

Reference	Number of cases	Number of classes	Ground truth	ROIs annotated method	Color Normalization	Modeling method	Result(AUC)
Echle et al. (33)	N=8,836	2	dMMR or MSI vs. pMMR	manually	Macenko method	ShuffleNet	Training:0.920 Validation:0.950(without color normalization) 0.960(after color normalization) Biopsy samples:0.780 AUPRC:0.790
Jiang et al. (28)	N=1,215	2	MSI vs. MSS	Densnet121 model automatic classification	Color augmentation	Densenet-IBM	Fully supervised:Cohort 1: 0.883; Cohort 2: 0.841; Cohort 3: 0.813; Cohort 4: 0.746 Weakly supervised:Cohort 1: 0.889; Cohort 2: 0.881; Cohort 3: 0.846; Cohort 4: 0.768
Yamashita et al. (57)	N=837(patient n=599)	2	MSI vs. MSS	automatic classification	Macenko method	MobileNetV2	Internal dataset: 0.931; External dataset: 0.779 Compared to pathologists: Model:0.865; Mean AUC performance of the five pathologists: 0.605
Bilal et al. (58)	N=499(patient n=502)	2	High mutation density vs. low mutation density; MSI vs. MSS; Chromosomal instability vs. genomic stability; CIMP-high vs. CIMP-low;BRAF mutation vs. BRAF wild-type;TP53 mutation vs. TP53 wild type; KRAS mutation vs. KRAS wild type	Resnet18 model automatic classification	NA	Resnet34	Multiple task prediction: Hypermutation:0.810; MSI: 0.860; Chromosomal instability: 0.830; BRAF:0.790; TP53: 0.730; KRAS: 0.600; CIMP-high status: 0.790
Schirris et al. (59)	N=360	2		automatic detection of tumor	Macenko method	DeepSMILE(from Self-supervised Heterogeneity-aware Multiple Instance Learning)	MSI: 0.870; HRD (homologous recombination deficiency): 0.810
Ghaffari Laleh et al. (60)	N=2,980	2	MSI vs. MSS	No annotation	Macenko method	Compared six methods	Classical weakly-supervised:Resnet:0.917; EfficientNet:0.930; ViT:0.906 Multiple-instance learning-based:MIL:0.709; AttMIL:0.880; CLAM:0.795
Cao et al. (61)	NA	2	MSI vs. MSS	manually	z-score normalization on RGB channels	Ensemble Patch Likelihood Aggregation(EPLA) model (Resnet18 was used for each patch prediction, PALHI and BoW pipelines integrated the multiple patch-level into WSI-level	Test: 0,885; External validation:0.850
Echle et al. (62)	N=8,343	2	MSI/dMMR vs. MSS/pMMR	No annotation	Macenko method	Resnet18	The highest cohort: 0.960; The lowest cohort:0.740 Biopsy samples: 0.890

The construction of most models relies on large sample datasets. Echle et al. (33) collected 8,836 HE-stained WSI of colorectal adenocarcinoma patients from five centers, including the TCGA database, to establish a deep learning classifier. To assess the impact of the number of training samples on performance, models were trained with samples ranging from 500 to 5500. The findings show that the robustness of the model increases as the number of training samples grows, reaching a stable level at 5000. More training data increases the number of features incorporated into the model, leading to better performance. Notably, this experiment also highlights that biopsy samples with limited tissue can be used to predict MSI. The classifier was tested using 1,557 biopsy samples, and the AUC was reduced to 0.780 compared with the surgical sample of 0.960. 2 years later, the same research team (62) established AI-based MSI/dMMR detectors based on surgical specimens, and the AUC of biopsy samples increased to 0.890.

In addition to predicting MSIs, there are models to predict polygenic states. For example, Bilal et al. (58) reported algorithms, based on Resnet34, to predict multiple gene expression status simultaneously, including the chromosome status, CpG island methylation, and BRAF, TP53, and KRAS gene mutational statutes. All models exhibited AUCs exceeding 0.900 at internal datasets. Still, all had decreased AUCs when validated on external datasets. A report (59) also establishes a model to predict APC, KRAS, PIK3CA, SMAD4, and TP53 gene mutations. Frozen and paraffin sections showed AUC values of 0.693-0.809 and 0.645-0.783, respectively, indicating the potential of deep learning in gene mutation prediction.

In summary, models can serve as an automatic screening tool to triage patients in predicting gene mutations, especially in MSI/MSS detection, ultimately resulting in significant cost and labor savings related to testing.

## 4 Recent advances of pathomics in CRC prognosis

### 4.1 Prediction of responses to neoadjuvant treatment

Neoadjuvant chemoradiotherapy is a common treatment modality for CRC and it has a vital role in improving surgery rates and survival in patients with resectable CRC (63). However, only 30% of patients achieve pathological complete response (pCR) (64). Some studies have demonstrated that radiomic features can predict the response to neoadjuvant chemoradiotherapy in preoperative CRC patients (65, 66). In 2020, the first paper using WSIs to predict the efficacy of neoadjuvant chemoradiotherapy was reported. Zhang et al. (67) used preoperative biopsy digital pathology images to predict the response to neoadjuvant chemoradiotherapy in patients with locally advanced rectal cancer. The authors extracted 104 texture features from selected tumor region tiles based on a machine learning approach and screened 17 potential predictors using the LASSO method. SVM-based classifiers distinguished these predictors. The AUCs of the classifiers were 0.887 and 0.797 for PR and non-PR at the tile level, and 0.930 and 0.877 for the model at the WSI level respectively. In the same year, Shao et al. (68) combined radiomic features with pathomics features to predict the efficacy of neoadjuvant therapy. They extracted 702 quantitative features from T2WI and ADC sequences, and together with a total of 770 image features extracted from WSIs, including pixel intensity, morphology, and nuclear texture based on the XGBoost method to construct the model radiopathomics signature (RPS), the accuracy of RPS reached 87.66%, with AUCs of 0.98 (TRG0), 0.93(≤TRG1), 0.84(≤TRG2), and the performance of this model is better than constructing the model based on MRI features, or pathological image features alone. One year later, the same team (69) reported another study focusing on the cell nuclei and the tumor microenvironment in pathology images. They used CellProfiler and VGG19 convolutional neural networks to extract 770 tumor cell nuclei features and 220 tumor microenvironment features, respectively. Combined them with 2,106 MRI image features to construct the prediction System (RAPIDS) model, which was further validated in a prospective study. The model has a high accuracy in predicting pathological complete response and an AUC of 0.812 in a prospective study. In addition, the combined model is significantly better than the single-modality prediction model.

In summary, AI has great potential in predicting the response to neoadjuvant therapy for colorectal cancer. By analyzing a large number of pathological image features and the potential correlation between them and treatment outcomes, these models can predict how patients will respond to neoadjuvant therapy while also providing patients with more accurate treatment recommendations.

### 4.2 Prediction of survival

As presented in **Table 3**, the pathological features extracted by AI to predict the prognosis of CRC are numerous and varied. In a study by Kather et al. (70), a tumor microenvironment-related prognostic factor was proposed for the prediction of CRC survival. Specifically, the tissues of CRC patients were first automatically classified into 9 categories at the tile level, including CRC epithelial cells, tumor-associated stroma, lymphocytes, debris, adipose tissue, background, mucus, smooth muscle, and normal colon mucosa, respectively. Using the univariable Cox proportional hazard model, 5 of the 9 categories were associated with poor outcomes: adipose tissue, debris, lymphocytes, smooth muscle, and tumor-associated stroma. The characteristics of these 5 tissue types were extracted and combined by the VGG19-based CNN model to establish the deep stroma score, which was an independent prognostic factor for overall survival in CRC (HR 1.99 [1.27-3-12], p=0.0028) using a multivariate Cox proportional hazard model. Zhao et al. (71) proposed a deep learning model for automatic tumor stromal ratio (TSR) quantification. Similar to Kather et al, they classified CRC patients’ tissues into 9 categories and trained a model based on the VGG-19 architecture. They found that TSR could be an independent prognostic factor in 2 independent cohorts of CRC patients, with stroma-low associated with a higher five-year survival rate. Subsequent incorporation of independent risks (stage and age) together to build a predictive model showed that the model demonstrated significant predictive power for patient prognosis with high accuracy and discrimination (ACC:0.759, C-index:0.721). Skrede et al. (72) developed a DoMore-V1-CRC classifier to predict cancer-specific survival in colorectal patients. These authors used the univariate and multivariate Cox proportion hazards model and Kaplan-Meier analysis to analyze the association of pathological features and pathological clinical variables with cancer-specific survival. They concluded that the pathological features extracted by the classifier could serve as strong predictors of prognosis and they can be used to complement established molecular and morphological prognostic markers. Similarly, Wulczyn et al. (73) developed a deep learning system (DLS) for predicting 5-year cancer-specific survival in grade II and III CRC. Significantly, the team generated 200 histological features based on clustered embeddings in a deep-learning image similarity model, which enabled the model to extract pathological features with human interpretability. The analysis reveals that the degree of tumor differentiation and the proportion of tumor stroma were the main features of DLS for predicting prognosis. Specifically, moderate to high grade tumors were associated with the high risk prediction by DLS, while low grade tumors and high stroma ratio were associated with low risk prediction of DLS.

**Table 3:** Literature overview of AI-based algorithms for CRC prognosis using histopathological images.

Reference	Number of cases	ROIs annotated method	Modeling method	Findings	Prognostic factors
Kather et al. (70)	N=909	VGG19 model automatic classification	VGG19	Cohort1:OS(overall survival): hazard ratio(HR): 1.99 [1.27-3.12] Cohort2:OS : HR:1.63[1.14-2.33]; CRC-specific OS : HR:2.29[1.5-3.48];relapse-free survival:HR:1.92[1.34-2.76]	Deep stroma score
Zhao et al. (71)	N=814	VGG19 model automatic classification	VGG19	Stroma-high associated with reduced OS, Cohort1: OS : HR:1.72[1.24-2.37] Cohort2:OS : HR:2.08[1.26-3.42];	Tumor-stroma ratio (TSR)
Skrede et al. (72)	N=2,473	DeepLab network automatic segmentation	DoMore v1	Cancer-specific survival:HR:3.84[2.72-5.43]; sensitivity:52%; Specificity:78%	Tumor area
Wulczyn et al. (73)	N=3,652	Inception-v3 model automatic classification	CNNs, similar to the design of MobileNet	Cohort1: 5-year disease-specific survival AUC:0.70 Cohort2: 5-year disease-specific survival AUC:0.69	Tumor area, tumor-adipose
Lin et al. (74)	N=1,686	VGG19 model automatic classification	VGG19	Cohort1:OS : HR:1.54[1.08-2.19]; Cohort2:OS : HR:1.36[1-1.84]; Cohort3:OS : HR:1.83[1-3.35];	Adipose tissue
Xu et al (75)	N=448	Resnet18 model automatic classification	Resnet 18, Resnet34, Shufflenet	Cohort1:progression-free survival(PFS):HR:0.004[0.0001-0.15]; Cohort2:PFS : HR:0.031[0.001-0.645]	Tumor-infiltrating lymphocytes
Wang et al. (76)	N=103	NA	DeepCon*vs.*urv model, Nomogram model	OS: AUC:0.86 DFS: AUC:0.875	Combing pathomics,radiomics features, immunoscore and clinical factors

In addition to studying the tumor stroma ratio, some studies have focused on the lipid microenvironment surrounding CRC. Lin et al. (74) trained the VGG-19 model to score adipose (ADI) tissue quantitatively in CRC and used Kaplan-Meier analysis to compare the OS of patients with high ADI to those with low ADI, and they found that the OS time was significantly lower in the high ADI group than in the low ADI group. In addition, tumor-infiltrating lymphocytes (75) can be used as a prognostic factor for CRC.

In summary, AI has shown potential in predicting the survival of colorectal cancer patients by analyzing not only the tumor region but also the tumor microenvironment. By using quantitative analysis techniques, AI can help identify important factors in the tumor microenvironment that can affect patient survival.

## 5 Integration of pathomics and other omics

A wealth of data is available during the actual diagnosis and treatment of CRC patients, ranging from radiology, pathology, colonoscopy, clinical data, and laboratory testing, to genomic information, each of which can provide information to assess the patient’s status. Given the enormous complexity of medical data, most of the data currently used to build AI models is monomodal. However, compared with monomodal algorithms, multimodal programs might help extract features from different perspectives, bring complementary information, and facilitate better decision-making. For example, radiological and pathological images provide microscopic and macroscopic information about the lesion tissue which can be combined to diagnose and stage CRC. Additionally, multi-modal data fusion is helpful to find the correlation and causality between different levels and to identify the characteristics that have prognostic or therapeutic significance. For example, by integrating pathological images, genome data, transcriptome data, and other data types, we can make molecular typing of tumors, and predict treatment response and survival (16, 77, 78). In CRC, studies have used radiomics and pathomics features in combination of clinical data to predict the treatment response (65, 68, 69) and survival (76). There are also studies (58) to investigate the correlation between gene expression changes and histomorphology, using genomic data and histological images to predict MSIs. Although the study of integrating pathomics and other omics with colorectal cancer has not been fully developed, it can be seen in the multimodal data fusion of artificial intelligence for other cancers.

Different strategies, such as connection-based, model-based, and transformation-based integration methods, can be employed for data fusion (79). Through such a multi-modal data integration analysis method, AI can assist researchers in comprehending the heterogeneity and complexity of tumor cells in greater detail. This, in turn, offers a stronger foundation for precise diagnosis and individualized treatment.

## 6 Challenges and perspectives

Studies have proved that with the continuous in-depth research of AI technology represented by deep learning in CRC, AI can aid pathologists in making more accurate and effective diagnoses, evaluating the therapeutic response, and predicting the prognosis before receiving treatment. In most published papers, researchers construct their models through transfer learning, which aims to first train the selected neural network model in the large dataset of the source domain, usually in the ImageNet Database (80), and then fine-tune it in the labeled pathological images to finally adapt the model to its task. Alternatively, some researchers may choose to train a deep network from scratch using domain-relevant images. The transfer learning model refers to the mode of learning new knowledge by leveraging existing related knowledge. This technique enables improved model performance and reduces computational costs by transferring similarities between existing and new knowledge. Conversely, training models from scratch entails establishing and training a model without any prior knowledge, offering simplicity but requiring substantial amounts of data and computational resources. Both models have their advantages and disadvantages, with transfer learning models not only saving training time and computing resources but also enhancing the model’s generalization ability and accuracy by incorporating knowledge and experience accumulated in other fields or tasks. Additionally, transfer learning models can tackle the scarcity and heterogeneity of medical data by improving the model’s representation ability with data from other sources. For example, a CNN trained on natural images can be used to extract image features, which can be fine-tuned or have the final classifier replaced to match the requirements of specific medical tasks. However, the transfer learning model does not apply in all situations. When the target domain differs significantly from the source domain, the negative transfer may interfere with the acquisition of accurate knowledge in the target domain. In such cases, training models from scratch can better adapt to specific tasks by designing the appropriate model structure and parameters according to the data and goals. Overall, transfer learning models are usually superior to models trained from scratch for medical image classification tasks. AlexNet, ResNet, VGGNet, and GoogleNet are among the most common and effective transfer learning models that have shown good results across various types, scenes, and objectives of medical image classification.

### 6.1 Data

Using AI algorithms to train models requires massive, multicenter, diverse, and high-quality data. With the ability to learn from vast amounts of data, these algorithms can offer new insights into the development of CRC, identify new predictive and prognostic factors, and facilitate individualized treatment plans. Numerous large publicly available databases of CRC histopathological images exist, including the TCGA database (81), the 2015 MICCAI Gland segmentation (GlaS) challenge dataset (82), colorectal adenocarcinoma gland (CRAG) dataset (83), Digestpath (84), and COMET dataset (85). However, such datasets are typically not labeled or annotated, and biopsy samples may result in lost morphological features due to the sampling method. For instance, the preparation process of colonoscopy biopsy specimens can squeeze some tissues, leading to changes in their original morphological characteristics. These defects can impair the training power of an AI algorithm, and limited training data can often lead to model overfitting. To address this issue, researchers (86, 87) may decide to either reduce the complexity of network architecture or procure additional training data. Data augmentation technology can also enhance the number of training samples in a limited dataset, improving the model’s overall performance (88). Pathology images have various data enhancement techniques, such as tissue classification (89), cell nucleus segmentation (90), gland segmentation (91), and prediction of microsatellite status (92), that can expand smaller datasets and improve their functionality. Furthermore, training models on synthetic images can produce similar results to authentic images (93, 94).

### 6.2 Ground truth annotation

The training of the model heavily depends on the manual assignment of pathology image labels for learning and classification. The gland is a critical component of colorectal tissue that has a typical round or elliptical shape and neat arrangement in normal samples, making manual labeling relatively simple. However, as cancerous tissues develop, the gland’s normal structure may become disrupted, resulting in irregular shapes and disorganized configurations. As a result, histological characteristics of such tissues typically show significant individual variations that are challenging to delineate manually (95). Pathologists often spend a considerable amount of time classifying and labeling CRC tissues, particularly tumor tissues that are challenging to identify and diagnose and require senior pathologists to identify and label them as part of the model’s training set to ensure machine learning accuracy. Unsupervised (96) or self-supervised learning (97) can alleviate these issues since they do not require explicit labeling, holding promise for overcoming these challenges.

### 6.3 Interpretability

Currently, the most effective AI algorithms for processing pathological images are deep learning based on neural convolution networks. These models may perform better than humans, but they have been questionable for the AI black-box problem (47, 49). The opacity of these models may be due to the inability of humans to perceive the decision-making pattern of machine learning algorithms (98). The deep neural network, for example, has thousands of neurons that allocate information and make decisions. As the number of network layers increases, the features extracted by the neurons become more abstract and incomprehensible to humans (99).

Some studies have used visualization methods to interpret the models to improve black-box transparency. Specifically, most researchers utilized heatmap (23, 33, 58, 100), and the attention mechanism (101–103) to visualize the features of histopathology images. These methods present the data using a heatmap overlaid on top of the original image, with darker colors signifying higher response and contribution of the corresponding region of the original image to the network model. These methods can help improve the transparency and feasibility of AI.

In summary, pathomics is a new tool that can comprehensively extract features and has the potential to improve the diagnosis of CRC. Moreover, it is increasingly important in determining the efficacy and prognosis of CRC treatment.

## Author contributions

BX and YW conceived and designed the study. YW, XX, YL, XL, and BL performed the reference analyses and wrote the manuscript. All authors contributed to the article and approved the submitted version.
